# Hydroxyapatite nanoparticle-modified porous bone grafts with improved cell attachment†

**DOI:** 10.1039/d3tb01839c

**Published:** 2023-10-19

**Authors:** Prachi Dhavalikar, Dana Jenkins, Natalie Rosen, Aparajith Kannapiran, Karim Salhadar, Orren Shachaf, Michael Silverstein, Elizabeth Cosgriff-Hernández

**Affiliations:** a Department of Biomedical Engineering, University of Texas at Austin 107 W. Dean Keeton, BME Building, Room 3.503D Austin Texas 78712 USA cosgriff.hernandez@utexas.edu +512-471-0616 +512-471-4679; b Department of Materials Science and Engineering, Technion–Israel Institute of Technology Haifa 32000 Israel

## Abstract

Emulsion-templated foams have displayed promise as injectable bone grafts; however, the use of a surfactant as an emulsifier resulted in relatively small pores and impedes cell attachment. Hydroxyapatite nanoparticles were explored as an alternative stabilizer to address these limitations. To this end, hydroxyapatite nanoparticles were first modified with myristic acid to generate the appropriate balance of hydrophobicity to stabilize a water-in-oil emulsion of neopentyl glycol diacrylate and 1,4-butanedithiol. *In situ* surface modification of the resulting foam with hydroxyapatite was confirmed with elemental mapping and transmission electron microscopy. Nanoparticle-stabilized foams displayed improved human mesenchymal stem cell viability (91 ± 5%) over surfactant-stabilized foams (23 ± 11%). Although the pore size was appropriate for bone grafting applications (115 ± 71 μm), the foams lacked the interconnected architecture necessary for cell infiltration. We hypothesized that a co-stabilization approach with both surfactant and nanoparticles could be used to achieve interconnected pores while maintaining improved cell attachment and larger pore sizes. A range of hydroxyapatite nanoparticle and surfactant concentrations were investigated to determine the effects on microarchitecture and cell behavior. By balancing these interactions, a co-stabilized foam was identified that possessed large, interconnected pores (108 ± 67 μm) and improved cell viability and attachment. The co-stabilized foam was then evaluated as an injectable bone graft including network formation, microscale integration with bone, push out strength, and compressive properties. Overall, this work demonstrated that *in situ* surface modification with nHA improved cell attachment while retaining desirable bone grafting features and injectability.

## Abstract 2

Hydroxyapatite nanoparticles and surfactant concentration in polyMIPE scaffolds affect microarchitecture and cell behavior. Co-stabilized polyMIPEs balance these interactions to achieve properties suitable for bone grafting applications.

## Graphical abstract

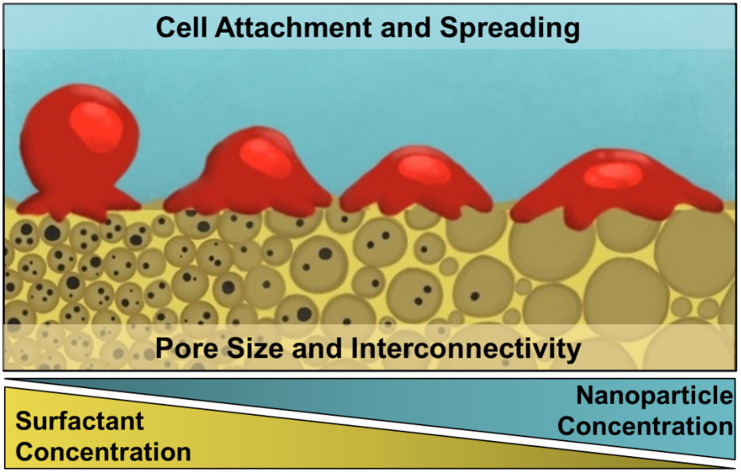

## Introduction

Large bone defects remain a significant clinical challenge and require surgical intervention to treat non-unions. Although autografts are the current “gold standard” for treatment, the limited amount of autograft that can be harvested has led researchers to develop synthetic bone grafts that can match its regenerative potential.^1–3^ Emulsion templating is a fabrication technique that generates porous scaffolds through the polymerization of the external phases of high internal phase emulsions (HIPEs, internal phase volumes of >74%) and medium internal phase emulsions (MIPEs, internal phase volumes of 40–74%).^4^ Numerous monomers and macromers have been used in the fabrication of polyHIPEs and polyMIPEs including styrene, acrylates, methacrylates, polyester diols and triols with diisocyanates, acrylated and methacrylated polyesters, various thiol–ene and thiol–yne pairs, among others.^5–7^ Our lab previously reported the fabrication of emulsion-templated scaffolds with suitable osteoconductivity, biodegradation, and compressive properties for bone grafting applications. These emulsions can be applied as injectable grafts to stabilize large defect sites or emulsion inks for 3D-printed bone grafts to match complex bone geometries.^6,8–10^

Emulsifiers in HIPEs and MIPEs are used to stabilize the emulsion by reducing the interfacial tension between two insoluble liquids and preventing droplet coalescence. Most commonly, surfactants (5–50% w/w) have been used in MIPE and HIPE formulations.^11^ Surfactant-stabilized emulsions yield scaffolds with highly interconnected porous structures that allow vascularization and metabolite transport, a critical process for tissue regeneration.^11–13^ However, surfactant-stabilized scaffolds typically have a relatively small pore size and surfactant can interfere with protein adsorption and cell adhesion on the scaffold surface.^14,15^ Lack of cell adhesion can slow healing and reduces healing outcomes.^16^ Removal of the surfactant after polymerization with Soxhlet extraction can be used to enhance cell attachment,^17,18^ but can lead to the collapse of the porous structure.^19,20^ Moreover, an injectable graft precludes post-fabrication processing of the scaffolds before implantation.^21,22^

**Fig. 1:**
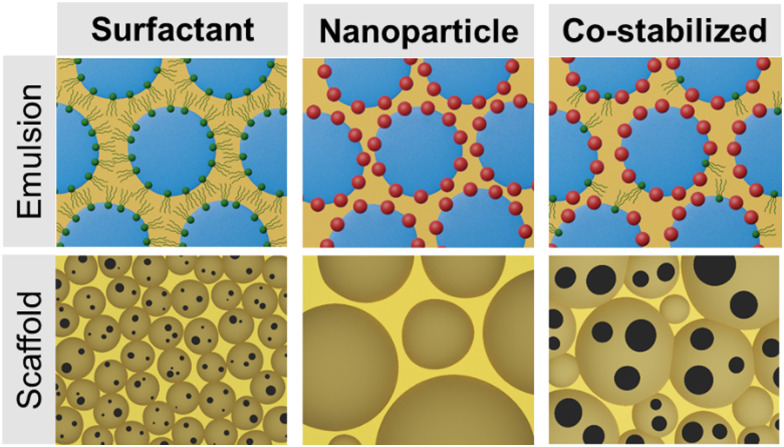
Schematic of emulsion stabilization mechanisms based on emulsifier type and hypothesized effects on emulsion-templated architecture. Surfactant-stabilized emulsions result in an open-pore structure after polymerization; whereas, nanoparticle-stabilized emulsions yield a closed-pore structure with larger pores. Co-stabilized emulsions with surfactant and nanoparticles can be used to template an open-pore foam with larger pores.

As an alternative to surfactants, Pickering emulsions use solid particles as emulsifiers.^23,24^ Since the 1990s, nanoparticle emulsifiers have gained considerable attention from researchers with potential applications in various fields, such as food, cosmetics, pharmaceuticals, and oil industries.^23^ Silica, clay, and starch nanoparticles are examples of particles used to form these emulsions and can be used to further modify the material properties such as conductivity or stimuli-responsiveness.^25–27^ For tissue-engineered scaffolds, Pickering emulsions offer control over cell-material interactions through *in situ* surface modification of the emulsion-templated graft, which permits direct injection into a defect site.^28^ To achieve efficient self-assembly at the pore surface, particle concentration, chemistry, size, shape, and surface roughness must be tailored to promote nanoparticle adsorption at the interface and stable emulsion formation.^29–33^ Once an appropriate formulation is identified that promotes stable emulsion formation, the resulting internal phase droplets are typically larger than corollary surfactant-stabilized emulsion. Once polymerized, these Pickering emulsions template larger, closed-pore structures that can limit cell infiltration. To address this limitation, prior research has successfully demonstrated the fabrication of foams with the large, open-pore architecture using a combination of nanoparticles and surfactant, Fig. 1.^34–37^

The goal of this study was to develop nanoparticle-stabilized emulsions to eliminate the use of surfactants and enable *in situ* surface modification of injectable bone grafts. Natural bone is a hybrid biomaterial composed of organic, like collagen, and inorganic compounds, including hydroxyapatite (HA).^38^ Research has demonstrated that natural and synthetic HA possess osteoconductive and osteoinductive properties and has been used clinically since the 1980s.^39–42^ Prior research in our laboratory demonstrated that the addition of HA nanoparticles (nHA) to a surfactant-stabilized HIPE had minimal effect on the osteogenic differentiation of human mesenchymal stem cells (hMSC).^7^ We hypothesized that the broad range of HA nanoparticle sizes used resulted in the depletion of the particles from the interface and preferential location in the pore wall rather than at the surface.^7^ Furthermore, surfactant-stabilized polyHIPEs displayed reduced cell attachment without removal of the surfactant. To address these issues, critical parameters, such as modulating nanoparticle hydrophobicity, size, and shape, were studied to understand nHA adsorption and emulsion stabilization mechanisms. The resulting polyMIPE scaffolds were characterized to highlight the effect of nHA incorporation on scaffold properties and hMSC behavior. Then to create an open-pore, surface-modified polyMIPE scaffold, we tuned PGPR and nHA concentrations of co-stabilized polyMIPE grafts. An increase in pore size (>100 μm) and improved cell attachment compared to the controls were used to select a candidate co-stabilized polyMIPE composition for evaluation as a bone grafting material. Overall, this work aims to build upon a fundamental knowledge of co-stabilized emulsion formation, demonstrate the benefits of *in situ* surface modification with nHA in our injectable bone grafts, and present an opportunity for the application of these emulsions for other fabrication platforms to broaden the available properties of scaffolds for bone grafting procedures.

## Materials and methods

### Materials

Polyglycerol polyricinoleate 4125 (PGPR) was donated by Paalsgard® (Denmark). All other chemicals were purchased from Sigma Aldrich unless otherwise noted. Neopentyl glycol diacrylate (NGDA) was filtered through an aluminum oxide column to remove the monomethyl ether hydroquinone inhibitor. The purified product was stored at 4 °C prior to polyMIPE fabrication.

### Surface modification of nHA

The surfaces of commercial hydroxyapatite nanoparticles (Aldrich, nanopowder, <200 nm particle size, 9.4 m^2^ g^−1^, ≥97%, synthetic) were modified with myristic acid. Myristic acid increases the hydrophobicity and amphiphilicity of the nanoparticles, allowing them to spontaneously migrate to the oil–water interface. The nHA surfaces were cleaned before modification through Soxhlet extraction in ethanol (ABS AR, Gadot group) for 24 hours and then dried for 20 hours in a vacuum oven at 100 °C.^43^ Myristic acid (*M*_w_ = 228 g mol^−1^, Sigma Aldrich) was dissolved in xylene (AR, Gadot) in a stirred reactor, and then the cleaned nHA was added. The reaction at 160 °C was performed overnight in a stirred reactor with reflux using a Dean–Stark apparatus to remove water. The myristic acid-modified nHA was washed with dichloromethane (AR-b, Bio-lab, Israel) using a centrifuge three times for 5 min each at 12 000 rpm and then dried overnight in a vacuum oven at 75 °C. The average myristic acid surface coverage on the nHA was evaluated using thermogravimetric analysis (TGA) in air from room temperature to 750 °C at 20 °C min^−1^ (2050 TGA, TA Instruments). The TGA mass loss was recorded as a function of temperature from unmodified nHA and from the five modified nHA batches. Nanoparticles were sieved to remove large aggregates prior to mixing with the organic phase of the MIPE for scaffold fabrication.

### PolyMIPE fabrication

PolyMIPE scaffolds were fabricated with redox initiation as described previously.^6^ Briefly, two organic phases were prepared – one containing benzoyl peroxide (1% w/w) as an initiator and one containing trimethylaniline (1% w/w) as a reducing agent. In addition to the redox components, each mixture was composed of NGDA (90% mol of total organic phase), 1,4-butanedithiol (BDT) (10% mol of total organic phase), and the stabilizer. Initial concentration of nHA was 8.5% w/w and PGPR surfactant was 5% w/v. Co-stabilized compositions varied the surfactant and nHA concentrations as indicated. Mixtures were spun at 2500 rpm using a Flaktek Speedmixer for 2.5 minutes to promote nanoparticle and initiator dispersion. After mixing the organic phase, aqueous calcium chloride solution (1% w/v) was added to the organic phase in three additions (68.5% v/v). Calcium chloride was used to limit Ostwald ripening prior to cure. MIPE compositions were prepared using an in-house modified emulsion mixer (ESI,† Fig. S1). For co-stabilized MIPEs, PGPR diluted in BDT (50% w/w) was added into the emulsion between the second and third water additions to allow for stable emulsion formation. Emulsions were mixed at the lowest speed in 25 second intervals using the modified emulsion mixer. Once a stable emulsion was formed, as confirmed visually, MIPEs were placed into a double-barrel syringe, and the two components were mixed upon injection through a static mixing head (Seltzer, Dental Supply). A subset of MIPEs (*n* = 3) were cured with a glass thermometer in solution to determine the peak exothermic temperature. All other MIPE specimens were placed in a 37 °C aluminum bead bath to facilitate crosslinking overnight.

### SEM analysis

PolyMIPE samples were vacuum dried for 24 hours to remove the water before the characterization of the pore architecture. PolyMIPEs from surfactant and co-stabilized MIPEs were placed under vacuum at room temperature. Nanoparticle-stabilized polyMIPEs were dried at 70 °C to increase the drying rate due to their closed-pore structure. Scanning electron microscopy (SEM, Phenom Pro, Nanoscience Instruments) was used to characterize the average pore size for each composition. Circular specimens from three polyMIPE samples were sectioned and fractured at the center. Specimens were sputter-coated with gold with a thickness of ∼4 nm and imaged to yield nine images. Pore size measurements were conducted by measuring the diameters of the first ten pores that crossed the median of each 300× magnification micrograph. Average pore sizes for each polyMIPE composition are reported. A statistical correction was calculated to account for the non-perfect spherical pores, *h*^2^ = *R*^2^ − *r*^2^, where *R* is the void diameter's equatorial value, *r* is the diameter measured from the micrograph, and *h* is the distance from the center of the pore.^44^ The average pore size values were multiplied by this correction factor, resulting in a more accurate description of the pore diameter.

### Transmission electron microscopy

Transmission electron microscopy (TEM) was utilized to determine the hydroxyapatite nanoparticle size and characterize the degree of aggregation. A set of three polyMIPEs was stabilized with the modified nHA using the previously described procedure. Briefly, polyMIPEs were impregnated with a methyl methacrylate (MMA) to maintain the structure of the pores during specimen sectioning. PolyMIPEs were submerged in MMA solution containing benzoyl peroxide (0.5% w/w) under a low vacuum. Polymerization of MMA was allowed to proceed for 24 hours in a convection oven at 75 °C. PolyMIPE specimens approximately 100 nm thick were prepared by ultramicrotomy (Leica EM UC7) from the composites. The TEM specimens were placed on standard copper grids, coated with a 3 nm thick layer of amorphous carbon, and viewed at 200 kV (FEI Technai T20 LaB6 TEM). Nanoparticle sizes were measured using FIJI software.^45^

### X-ray energy dispersive spectroscopy

PolyMIPE specimens were prepared as described above for SEM analysis. Elemental mapping of the samples was performed in a high-resolution Apreo 2 scanning electron microscope (ThermoFisher Scientific) at 20 kV using X-ray energy dispersive spectroscopy (EDS). The resulting calcium and carbon elemental mappings were reported.

### Hydroxyapatite at pore surface

Alizarin red staining (ARS) was performed to determine the presence of calcium-rich hydroxyapatite nanoparticles at the surface of the pore foams. Foams were cut into 8 mm by 1 mm disks and dried overnight. Dry samples were incubated in ARS (2% w/v) for 5 minutes. Scaffolds were washed with deionized water to remove excess stains and photographed under an optical microscope.

### Integration with bone

The ability of the co-stabilized polyMIPE to be injected and cured in a bone defect was evaluated using an *ex vivo* bovine rib model. Briefly, cylindrical voids 1 cm in diameter and 0.5 cm in depth were created in the bone with a Dremel rotary tool and MIPE was injected into the defect using a double barrel syringe with mixing head. After curing in the bead bath, the bone with polyMIPE was sectioned and imaged using a scanning electron microscope as previously described.^10^

### Compressive mechanical properties

The polyMIPE compressive properties were tested with an Instron 3300, equipped with a 1000-N load cell. The polyMIPEs were cured in 15 mL tubes at 37 °C for mechanical testing. Each specimen was sectioned into three disks with a 3 : 1 diameter-to-height ratio, yielding heights of ∼15 mm, using an Isomet saw (Buehler) and compressed at a strain rate of 50 mm s^−1^. The compressive modulus was calculated from the slope of the linear region after correcting for zero strain, and the compressive strength was identified as the stress at the yield point or 10% strain, whichever occurred first. Average moduli and strength of three disks were reported.

### Gel fraction

The gel fraction was measured gravimetrically to evaluate the extent of network formation. After polyMIPEs were cured for 24 hours, samples were sectioned into 8 mm × 1 mm disks. The mass was recorded for each specimen after drying under vacuum for 48 hours, incubating in dichloromethane (DCM) at 1 mL/10 mg of the sample for 24 hours, and vacuum drying again until a constant mass was achieved. The final weight divided by the initial weight was assessed as the gel fraction and corrected for the mass of the surfactant that would dissolve in the DCM, if applicable.
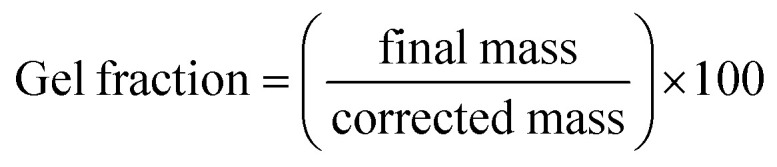


### hMSC culture

Cell studies used human mesenchymal stem cells hMSCs purchased from Texas A&M Health and Science Institute and cultured *in vitro* with Minimum Essential Medium with alpha modification (αMEM) with 16.5% fetal bovine serum (FBS), 1% l-glutamine, and 1% penicillin streptomycin solution. All experiments were performed with cells at passages 4–6. A standard LIVE/DEAD assay kit (Molecular Probes) was used to determine the effects of either surfactant or nanoparticle as an emulsifier on cell viability. PolyMIPE specimens (8 mm diameter × 1 mm height) were prepared for cell seeding: ethanol wetting ladder and overnight media incubation in PBS at 37 °C. Teflon weights were used to hold polyMIPE specimens in the well and the cell suspension was added directly to pre-wetted polyMIPE specimens at a density of 20 000 cells per well. Live/dead staining was conducted at specified time points. For LIVE/DEAD analysis, cells were washed with PBS and stained with 2 μM calcein-AM (live) and 4 μM ethidium homodimer-1 (dead) for 30 min at 37 °C. Images of each specimen were obtained through raster patterning using a fluorescence microscope (Nikon Eclipse TE2000-S).

### Statistical analysis

The data are displayed as mean ± standard deviation for each composition. An analysis of variance (ANOVA) comparison was used for multiple composition comparisons with Tukey's multiple comparisons to analyze the significance of the data. A Student's *t*-test was performed to determine any statistically significant differences between compositions. All tests were conducted at a 95% confidence interval (*p* < 0.05).

## Results and discussion

Several parameters affect the formation of a stable water-in-oil emulsion that is used to template the resulting foam after polymerization of the continuous phase. The hydrophobicity and viscosity of the monomer/macromer, emulsifier concentration and chemistry, and shear forces applied during mixing.^46,47^ Following a series of scouting studies to identify requisite organic phase characteristics, NGDA and BDT monomers were selected based on stable Pickering MIPE formation.^46^ Viscosity of the organic phase was the first challenge in introducing the internval aqueous volume. In Pickering emulsions, high viscosity of the continuous phase can prevent nanoparticle diffusion and adsorption at the interface.^48–50^ Tsabet *et al.* demonstrated that at oil phase viscosities above 485 mPa s (485 cP) droplet size distribution increased significantly, and emulsions were highly unstable, likely due to dampened nanoparticle movement and attachment at the oil–water interface.^48^ Slower particle adsorption kinetics likely affect stabilization when the initial attachment force of the particle is lower than the breakage force. In a highly viscous medium, shear allows for droplet formation but slow nanoparticle movement does not sufficiently stabilize the new interface. Adsorption also depends on capillary forces, and high organic phase viscosity can hinder film drainage preventing nanoparticle-nanoparticle contact.^51^ As a result, particles can detach rendering any generated droplets less resistant to coalescence.^48,52^ Furthermore, it is possible that due to the higher viscosities, the overall emulsification process is also affected, leading to a reduced interface generation capacity. An organic phase based on NGDA and BDT provided values within the range of the target viscosities, *η* = 6–7 cP. We also investigated the effect of continuous phase hydrophobicity on nanoparticle adsorption and emulsion formation. Similar effects of oil phase hydrophobicity affecting nanoparticle adsorption from changing particle wettability have been observed in other studies. Frelischowska *et al.* demonstrated that tuning hydrophobicity of the continuous phase by a mixture of oils impacted emulsion stability. As more hydrophobic oils were mixed in, an increase in droplet size distribution was observed, indicating in a loss of emulsion stability.^29^ In addition to requisite viscosity, the NGDA and BDT system provided appropriate hydrophobicity (log *P* = 2.0–2.4) for stable emulsion formation.^46^ It should be noted that these Pickering emulsions required a different mixing setup than our previous surfactant-stabilized emulsions. We previously utilized a dual asymmetric centrifugal mechanism (Flaktek SpeedMixer); however, nanoparticle-stabilized emulsions were only formed with hand-shaking. To replicate this motion in a reproducible manner, we modified a reciprocating saw to achieve the requisite mixing action for emulsion formation. Although emulsification was successful, emulsions maintained limited stability resulting in phase separation within one hour, indicating possible nanoparticle desorption from interface and droplet coalescence.

### Surface modification of nHA with myristic acid

**Fig. 2:**
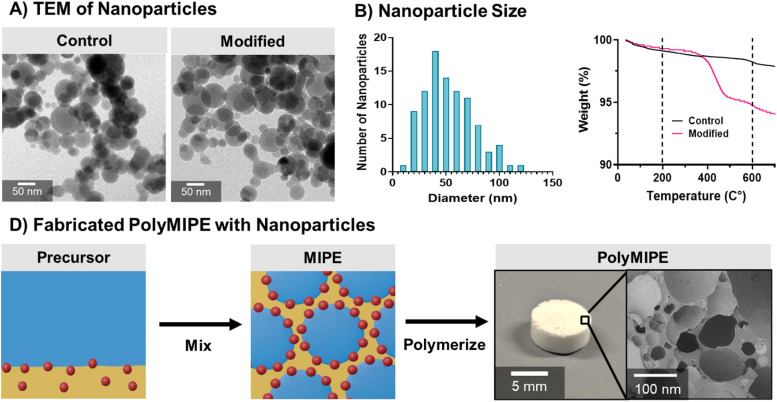
Developing nanoparticles capable of stabilizing MIPEs. TEM of the hydroxyapatite nanoparticles before and after surface modification (A), histogram of nanoparticle size distribution (B), and representative TGA curves (C) of myristic acid-modified nanoparticles compared to an unmodified control. Scheme of modified nanoparticles being used as an emulsifier to fabricate a polyMIPE scaffold (D).

Size, shape, and wettability were evaluated to identify a HA nanoparticle suitable to stabilize our selected polyMIPE formulation. HA is found in the inorganic component of bone and contributes significantly to the bone's strength and toughness.^53,54^ We selected synthetic nHAs to improve the translation of the nanoparticle-stabilized emulsions to bone tissue engineering applications. Commercially available hydroxyapatite before and after modification exhibited a spherical shape (Fig. 2A). It has been well-documented that spherical particles can stabilize emulsions.^55–57^ However, it has been demonstrated that nanorods or other shapes with higher aspect ratios have increased stability compared to emulsions stabilized by spherical particles.^58–60^ The efficiency of how the particles aggregate at the interface directly impacts stability. Particles of different shapes could be explored in the future to improve the stability of the emulsion. The size of the modified nanoparticles ranged from 13 to 125 nm, with a mean of 53 nm (Fig. 2B). We initially attempted nanoparticle formation with particles ranging up to 250 nm (data not shown), but they did not yield stable emulsions. The size of the particles can affect the emulsion stability and resulting internal phase droplet size.^32,56^ Qi *et al.* found that smaller poly(d,l-lactic-*co*-glycolic acid) nanoparticles (∼330 nm) exhibited more efficient packing at the droplet interface and less coalescence than the tested larger particles (620 nm and 1150 nm).^61^ Our study further supports that a smaller and more homogenous size nanoparticle population demonstrated improved emulsion stability.

Nanoparticle adsorption at the interface is a balancing act, and a key factor is the nanoparticle wettability, the contact angle at the oil–particle–water interface.^62,63^ Wetting the particles in one phase more than the other can hinder migration to the interface affecting emulsion formation.^50^ The use of nHA as an emulsifier also provides an opportunity for *in situ* surface modification of the pore walls to direct cell behavior, specifically promoting osteogenic phenotypes in MSCs.^41^ HA is naturally hydrophilic; therefore, to stabilize the water-in-oil emulsions, a hydrophobic surface modification was required. To achieve partial wettability of the nanoparticles, the nanoparticles were coated with myristic acid. Myristic acid was selected for its hydrophobicity and cytocompatibility and has previously been used to coat ceramics and hydroxyapatite to increase hydrophobicity.^64^ After coating the surface of the nanoparticle with the myristic acid, TGA analysis was used to confirm the successful modification. The mass loss between 25 and 200 °C was associated with water evaporation and the degradation of organic contaminants. The mass loss between 200 and 600 °C was associated with the degradation of myristic acid (Fig. 2C). The average coverage of nHA by myristic acid (moles per area) was calculated from the myristic acid mass loss, molecular weight, and the nHA specific surface area of 9.4 m^2^ g^−1^ (given as the minimal specific surface area in the specifications). The nHA batches with surface coverages at 17–38 μmol m^−2^ (3.5–8.2% mass loss) were used to make stable polyMIPEs (Fig. 2D). No phase separation was noted for extended periods, indicating successful nanoparticle-based emulsion stability. Surface coverages outside of this range batch did not form stable emulsions, indicating that the nHA hydrophobicity was not suitable for adsorption at the oil–water interface. Although there are reports of using hydroxyapatite to fabricate surfactant-free polyHIPEs, previous studies required the use of organic solvents to dissolve polymers to produce the continuous phase.^28,34,65^ To the best of our knowledge; there are no reports of solvent-free fabrication of emulsion-templated scaffolds stabilized by hydroxyapatite nanoparticles. The solvent-free fabrication used in these studies permits the system to be used as an injectable bone grafting platform.

### Characterization of nanoparticle-stabilized polyMIPE scaffolds

**Fig. 3:**
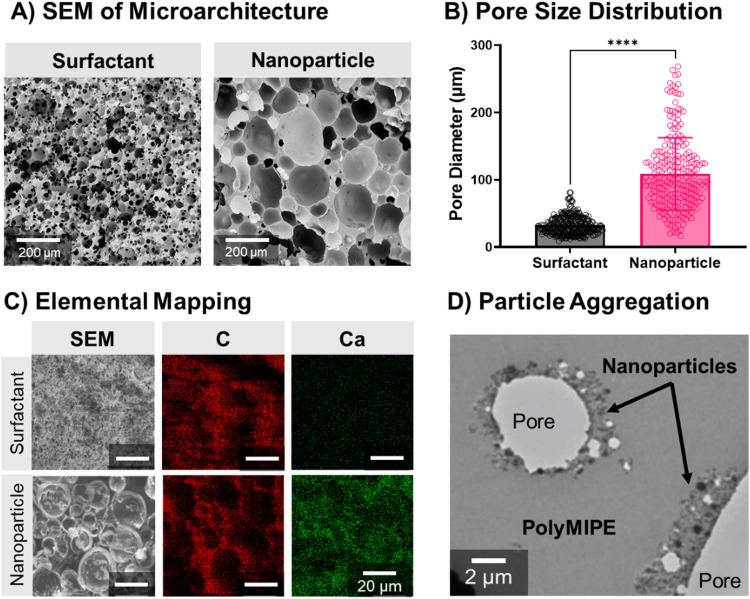
Representative SEM of surfactant *versus* nanoparticle-stabilized polyMIPEs (A) and calculated pore sizes for each composition (B). Nanoparticle localization at the pore surface was confirmed with carbon and calcium mapping with EDS (C) and TEM of the polyMIPE wall structure with the nanoparticles preferentially located around the voids (D). The * represents the statistical difference between groups (*p* < 0.05).

SEM characterization of the nHA-stabilized polyMIPE scaffolds microarchitecture indicated an increase in size and size distribution as compared to the surfactant-stabilized scaffold control (Fig. 3A). The average pore size for nanoparticle-stabilized polyMIPEs was 108 ± 67 μm compared to 34 ± 18 μm for the surfactant-stabilized control (Fig. 3B). The nHA spontaneously migrate to the oil–water interface to act as an emulsifier and results in the nHA assembled at the pore surface after polymerization.^30,66^ This surface modification was confirmed with EDS, ARS and TEM. Elemental mapping in Fig. 3C demonstrates increased calcium of nHA stabilized foams as compared to surfactant-stabilized foams. Calcium is present to a lesser extent at the pore surface in surfactant-stabilized polyMIPEs, which was attributed to the calcium chloride in the aqueous phase of the emulsion. This was also confirmed with ARS assay confirmed that the HA was exposed at the pore surface to direct cell–material interactions ESI,† Fig. S2. Fig. 3D shows a representative TEM micrograph of the pore surface and interior strut wall from a 8.5% w/w nanoparticle-stabilized polyMIPE with nHA preferentially at the surface. Together these studies verify that the particles form an *in situ* surface modification, which can be utilized to improve cell–material interactions and direct cell behavior. Successful *in situ* surface modification of the polyMIPE opens this platform to a breadth of surface modifications to tailor the material for other applications.

### Cell behavior on nanoparticle-stabilized polyMIPEs

**Fig. 4:**
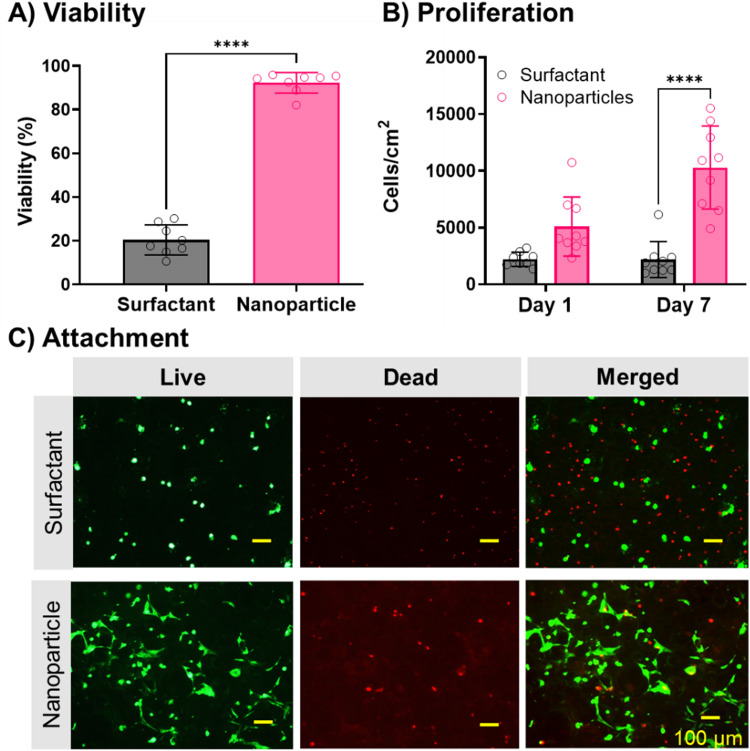
Effect of emulsifier on hMSC viability (A), proliferation (B), and attachment (C). Nanoparticle-stabilized scaffolds demonstrated a significant difference in cell behavior compared to surfactant-stabilized scaffolds. All data shows individual data points with average ± standard deviation for *n* = 7–10. The * represents the statistical difference between groups (*p* < 0.05).

Once nanoparticle-stabilized polyMIPEs were successfully fabricated, cell attachment to the scaffolds was evaluated without additional cleaning. Attachment, viability, and proliferation of hMSCs on the scaffolds was investigated. Initial hMSC viability testing demonstrated a significant increase in cell viability with 92 ± 6% cell viability on nanoparticle-stabilized grafts as compared to 19 ± 6% on surfactant-stabilized (Fig. 4A). Proliferation over seven days was quantified for both scaffolds, with a significant increase in cell count on the nanoparticle-stabilized polyMIPEs (day 1: 5090 ± 2610 cell per cm^2^, day 7: 10 290 ± 3660 cell per cm^2^) (Fig. 4B). Cells seeded on surfactant-stabilized polyMIPEs demonstrated no significant change between day 1 and 7 (day 1: 2210 ± 620 cell per cm^2^, day 7: 2180 ± 1580 cell per cm^2^). An increase in cell attachment and spreading can be noted from representative images of hMSC attachment in Fig. 4C. Cells seeded on the nanoparticle groups exhibited increased spreading; whereas, cells on the surfactant-stabilized scaffolds exhibited minimal cell attachment. These results were expected as the nanoparticle-covered surface provides the osteoconductive properties of HA that is known to enhance bone regeneration.^67–72^ Cells cannot attach and spread on the surface of biomaterials without ligands for integrin binding, primarily provided by serum protein adsorption to synthetic materials.^73^ We hypothesize that the surfactant interferes with this protein adsorption; whereas, the nHA acts to increase surface roughness and promote this protein adsorption.^67,69^ hMSCs are adherent cells and weak adhesion to the scaffold surface affects proliferation and differentiation, as seen for surfactant-stabilized polyMIPEs.^74^ Given that directing desired cell behavior on synthetic grafts is a great challenge, the evidence in cell viability and proliferation is promising for application in tissue engineering. The poor cell spreading, viability, and proliferation on the surfactant-stabilized scaffolds further support our aim of creating a scaffold with reduced surfactant concentrations to improve cell-to-material interactions.

### Investigation of pore-opening with co-stabilization

Although the nanoparticle-stabilized polyMIPEs demonstrated the desired increased pore size and cell behavior, the closed pore morphology would limit tissue integration. A consequence of using nanoparticles to stabilize emulsions is the closed-pore foam structure.^24,33,75^ The need for relatively full nanoparticle coverage of the droplet surfaces produces relatively large droplets, and therefore, relatively thick monomer films between the droplets. As stated previously, nanoparticle adsorption at the interface produces a layer that encases the internal phase droplets. The rigid layer acts as a barrier because nanoparticles bridging between closely packed droplets limits film thinning and subsequent interconnect formation. However, an interconnected structure is critical for tissue engineering applications because it enables cell infiltration and nutrient transport.^76^ We next explored using a combination of nanoparticle and surfactant emulsifiers to introduce interconnectivity to the polyMIPE foams.^36,77,78^ Synergistic stabilization using both emulsifiers is a dynamic process because added surfactant can adsorb onto the nanoparticle surface, changing the wettability.^36^ Based on previous work, there are differing perspectives on how this affects emulsion formation and stabilization. Some groups have hypothesized that this can still promote nanoparticle attachment, and the surfactant allows for sufficient film thinning and pore opening during polymerization.^78–80^ Other researchers have suggested that changes to the particle wettability result in particle disaggregation from the interface, causing a reduction in the continuous phase viscosity and a reduction in film thickness that enables interconnect formation.^36^

**Fig. 5:**
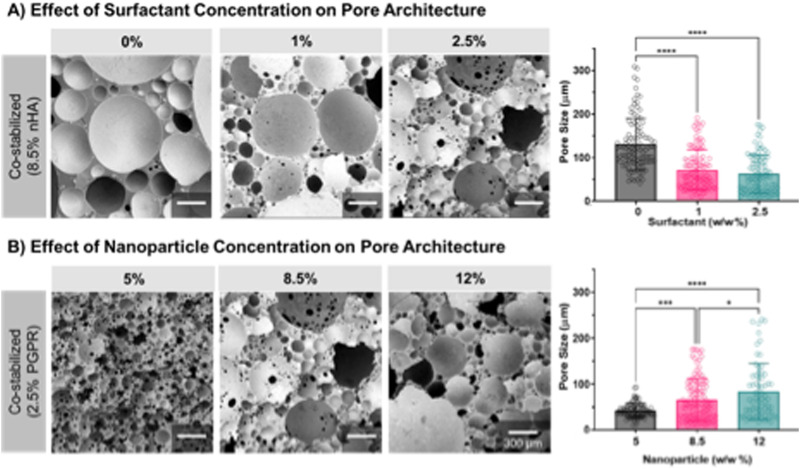
Effect of surfactant (A) and nanoparticle (B) concentrations on polyMIPE pore size and pore throat presence determined using SEM imaging. Representative images of each group are displayed. All data shows individual data points with average ± standard deviation. The * represents the statistical difference between groups (*p* > 0.05).

In these studies, preliminary attempts investigated emulsion formation by adding surfactant and nanoparticles into the continuous phase before water additions. Stable MIPE formation was not observed, suggesting surfactant affected nHA wettability. Similar results were observed in prior studies.^78^ We next attempted adding surfactant after forming the MIPE with nanoparticles. MIPEs maintained good stability, but an interconnected morphology was not observed after cure, as previously reported by Ikem *et al.*^36^ The closed-pore structure was likely due to insufficient surfactant, and we did not investigate higher concentrations similar to Ikem *et al.* due to our preliminary studies that highlighted the effect of surfactant on cell attachment. Therefore, we next investigated whether modifying the timing of the surfactant addition would enable stable MIPE formation with hierarchical porosity. We hypothesized that adding the surfactant before the last water addition would enable direct surfactant adsorption at the interface. If the bulk of the nHA has been used to stabilize the first two water additions, adding surfactant afterward would also limit nanoparticle-surfactant interactions. Upon investigation, this method enabled stable MIPE formation and the formation of an interconnected morphology after cure. Scanning electron micrographs of polyMIPEs from emulsions stabilized at different nanoparticle and surfactant concentrations are shown in Fig. 5. In the first study, the nanoparticle concentration was fixed at 8.5% w/w of the monomer since control MIPEs stabilized at this concentration were stable during polymerization. As the amount of surfactant increased, interconnect formation was observed, with the 2.5% w/w surfactant composition maintaining a more interconnected structure than the control, Fig. 5A. In a second study, porous foams were also fabricated at different nanoparticle concentrations while maintaining the surfactant concentration constant at 2.5% w/w PGPR. Compared to the 5% w/w nHA composition, interconnectivity was greatly reduced at 8.5% w/w/HA, Fig. 5B. Despite the surfactant, the 12% w/w nHA composition maintained a closed-pore architecture akin to the nanoparticle-only control. Although an open-pore structure was obtained, a corollary decrease in the overall scaffold pore size was observed. Increasing the surfactant concentration increased film-thinning between the droplets, allowing for greater interconnect formation. High shear from the mixing likely caused the reduction in the droplet size, specifically for the surfactant-stabilized interface.

The surfactant likely serves as the primary stabilization mode, as observed from the small pores and highly interconnected foam structure at lower nanoparticle concentrations. The lack of interconnects may result from a thinner nanoparticle layer between adjacent droplets and overall better dispersion of surfactant from the lack of nanoparticles in the emulsions. When the nanoparticle concentration increases, there is likely more nHA aggregation at the interface, producing robust bridging networks that are less permeable to the surfactant. As a result, this likely prevented sufficient surfactant adsorption at the interface to enable interconnect formation.

### Evaluation of surfactant and nanoparticle concentration on cell behavior

**Fig. 6:**
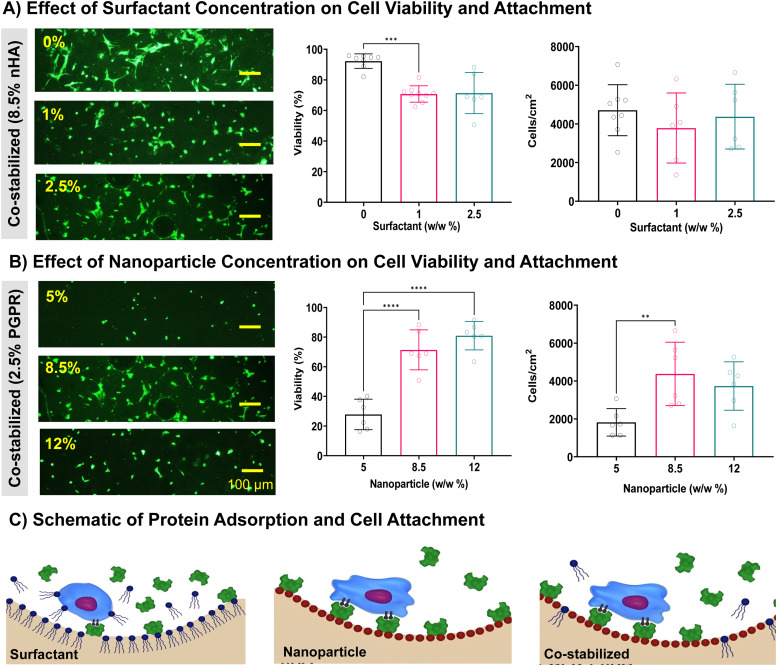
Effect of surfactant (A) and nanoparticle (B) concentration on hMSC viability and attachment on polyMIPE surfaces. Representative images of hMSC stained with calcein AM are displayed. Schematic of hypothesized mechanism of nanoparticles promoting protein adsorption compared to surfactants (C). All data shows individual data points with average ± standard deviation for *n* = 6–9. The * represents the statistical difference between groups (*p* < 0.05).

Previously we demonstrated that polyMIPEs stabilized with nanoparticles had improved cell attachment compared to the surfactant-stabilized scaffolds. To achieve an improved bone grafting material, the concentrations of surfactant and nanoparticles must allow an open-pore architecture without sacrificing cell viability and attachment. Concentrations of surfactant and nanoparticles were the same as those evaluated in Fig. 6. First, we determined the effect of increasing surfactant concentration in the polyMIPE scaffold (Fig. 6A). All samples evaluated were fabricated with 8.5% w/w nHA with 0%, 1%, or 2.5% w/w surfactant. Cell attachment was similar between groups; however, 1% and 2.5% w/w surfactant groups had a small but significant decrease in 24 hour viability as compared to the control without surfactant. Although there was no statistical difference in cells attachment, the decrease in viability and decreased cell spreading (as seen in representative images) suggest a potentially negative effect of the surfactant. Nonionic surfactants, like PGPR, have produced a reduction in protein adsorption, a key mechanism that enables cell attachment on biomaterials without innate biological cues. Dixit *et al.* observed that several nonionic surfactants reduced Fc-fusion protein adsorption when introduced before or with the protein solution.^81^ Similarly, Kapp *et al.* demonstrated that the nonionic surfactant polysorbate 80 inhabited adsorption of monoclonal antibodies when surfactants were pre-exposed to surfactant.^82^ These groups proposed that the lack of protein adsorption on the surface may be due to the stabilizing effects of the surfactant/protein interaction, which reduces the interfacial affinity of the protein to the polymer surface. Next, the role of nanoparticles on hMSCs cell attachment and viability was investigated (Fig. 6B). This study maintained a constant surfactant concentration of 2.5% w/w due to its more open-pore architecture, combined with 5%, 8.5%, and 12% w/w nanoparticles. Scaffolds with 5% w/w nanoparticles demonstrated significantly decreased cell attachment and viability than 8.5% and 12% w/w nanoparticle samples. There was no significant difference between 8.5% and 12% w/w, suggesting that the beneficial effects of the nanoparticles have a limit. The nHA has been shown to promote protein adsorption and cell attachment in other biomaterials.^83^ The high surface area most likely causes the increased protein adsorption per volume, allowing more proteins to adsorb to the surface. Several groups demonstrated that the nano-topography of the hydroxyapatite or calcium phosphate nanoparticles is critical in controlling protein adsorption behavior.^84,85^Fig. 6C depicts the effects of emulsifier selection (surfactant, nanoparticle, or co-stabilized) on protein adsorption schematically. It could be beneficial to further investigate the protein adsorption on the surface of nanoparticle-stabilized and surfactant-stabilized polyMIPEs in future studies to further elucidate the drive behind the improved biocompatibility. In summary, the improved cell attachment combined with co-stabilized pore architecture guided us to select polyMIPEs fabricated with 2.5% w/w PGPR and 8.5% w/w nHA for further evaluation as a bone grafting material.

### Evaluation of co-stabilized MIPEs in bone grafting applications

**Fig. 7:**
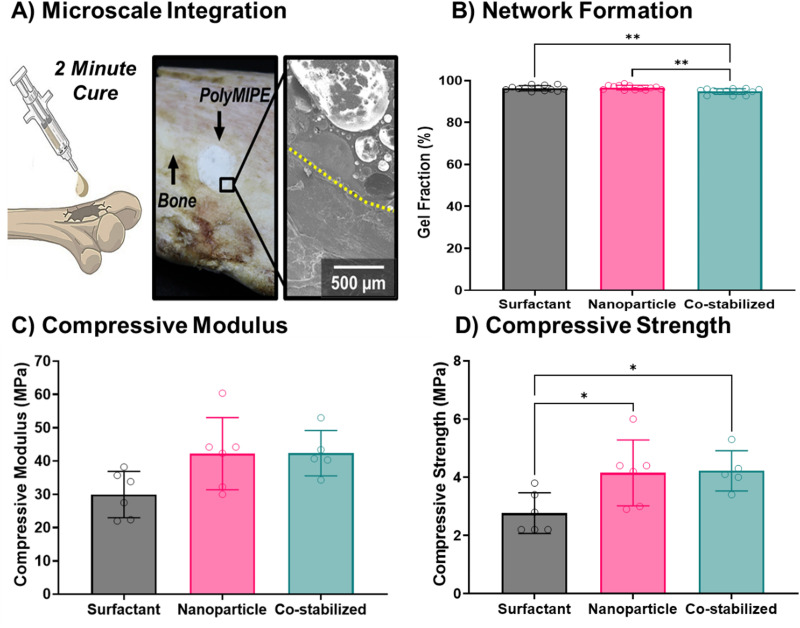
Evaluation of co-stabilized polyMIPE properties for bone graft applications. Demonstration of the co-stabilized MIPE as an injectable bone graft with microscale integration in bovine rib bone (A). The extent of network formation was determined using gel fraction (B). Compressive mechanical properties were observed for modulus (C) and strength (D). All data shows individual data points with average ± standard deviation for *n* = 5–13. The * represents the statistical difference between groups (*p* < 0.05).

To assess the potential of this new scaffold formulation as bone grafts, we assessed the microintegration with bone, network formation, and compressive mechanical properties of co-stabilized polyMIPEs. A seamless interface between the grafting material and native bone promotes mechanical stabilization and bone growth for an injectable bone grafting system.^86^ The co-stabilized MIPE was injected into a defect in a bovine bone and the MIPE cured within 2 minutes post-initiation and reached a maximum exothermic temperature of 39 °C (data not shown). Fig. 7A demonstrates that the co-stabilized polyMIPE bone graft seamlessly interfaces with the surrounding cortical bone. The localization of the nanoparticles at the pore surface was verified using ARS staining and EDS as previously done with surfactant and nanoparticle-stabilized polyMIPEs (ESI,† Fig. S2 and S3). Next, the extent of network formation of the polymer scaffolds was determined by the gel fraction (Fig. 7B). All the polyMIPE groups tested were demonstrated to have an average gel fraction ≥95%. The co-stabilized polyMIPEs gel fractions were 95 ± 1%, allowing for an injectable scaffold without post-fabrication modifications. Unreacted acrylate monomers have been shown to impact cell viability negatively.^87^ Nanoparticle-stabilized foams, in general, maintain gel fractions >90%, as demonstrated by other researchers, further confirming that this method of stabilization does not hinder polymerization and network formation.^22,33^ Finally, mechanical testing was performed (Fig. 7C and D). Representative stress–strain curves for each group can be viewed ESI,† Fig. S4. The nanoparticle-stabilized and co-stabilized polyMIPEs displayed a small increase in compressive modulus and statistically significant increase in compressive strength as compared to the surfactant-stabilized polyMIPEs. This was attributed to nanoparticle reinforcement of the pore walls and the closed-pore structure.^35^ The compressive properties of the co-stabilized polyMIPEs (modulus = 41.6 ± 6.4 MPa, strength = 3.6 ± 0.5 MPa) are similar to trabecular bone.^88^ Collectively, these results demonstrate the potential of the co-stabilized polyMIPEs as a bone grafting material.

Several research groups are pursuing emulsion-templated scaffolds for tissue regeneration. The co-stabilized polyMIPEs developed here provide several notable advantages over prior grafting materials in terms of pore size (>100 microns), *in situ* surface modification with nHA, favorable hMSC cell attachment, and suitable compressive properties for bone grafting. Multiscale porosity has been achieved by developing emulsion inks to address the relatively small pore size of other polyester-based approaches by our group and others.^8,89,90^ Dikici *et al.* utilized thiolene chemistry to fabricate polycaprolactone-based polyHIPEs with a broad range of pore sizes and compressive properties; however, the preparation of the emulsions included solvent which precludes its use as an injectable scaffold.^91^ Naranda *et al.* reported large pore sizes of polyHIPE scaffolds (50–170 microns) fabricated from tetrakis-3-mercaptopropionate and divinyladipate.^92^ These scaffolds displayed excellent potential with compressive properties suitable for cartilage tissue engineering. Future studies are planned to increase the interconnect size and perform cell infiltration studies and osteogenic differentiation studies prior to proceeding with evaluation in orthotropic animal models.

## Conclusion

A co-stabilized polyMIPE scaffold was developed with modified HA nanoparticles that demonstrated strong potential as an injectable bone graft. The effects of the nanoparticle and surfactant concentrations on the pore architecture and cell-growth behavior were evaluated. A candidate formulation of 8.5% w/w nHA and 2.5% w/w PGPR was determined for the co-stabilized polyMIPE that demonstrated the requisite properties for injectable bone grafting applications, including integration at the defect site, network formation, and the desired mechanical behaviors. Further research is required to evaluate the osteogenic differentiation of hMSCs to determine the scaffold's osteoinductive properties. Overall, this work showcases the ability to fabricate co-stabilized emulsions for *in situ* surface modification of polyMIPE scaffolds. These findings can guide the development of other biocompatible emulsion-templated scaffolds that promote cellular interactions while maintaining their architecture and properties.

## Author contributions

Prachi Dhavalikar – conceptualization, data curation, formal analysis, investigation, methodology, project administration, supervision, validation, visualization, writing – original draft. Dana Jenkins – conceptualization, data curation, formal analysis, investigation, methodology, project administration, supervision, validation, visualization, writing – original draft, writing – review & editing. Natalie Rosen – data curation, formal analysis, investigation, methodology, writing – original draft. Aparajith Kannapiran – formal analysis, investigation, validation, visualization. Karim Salhadar – formal analysis, investigation, validation, visualization. Orren Shachaf – formal analysis, investigation, validation. Michael Silverstein – conceptualization, funding acquisition, methodology, resources, project administration, supervision, writing – review & editing. Elizabeth Cosgriff-Hernández – conceptualization, funding acquisition, methodology, resources, project administration, writing – review & editing, supervision, visualization.

## Conflicts of interest

There are no conflicts to declare.

## Supplementary Material

TB-011-D3TB01839C-s001
