# Supplementary material for: Hydroxyapatite nanoparticle-modified porous bone grafts with improved cell attachment
Source: J Mater Chem B. 2023 Oct 19;11(44):10651–64. doi: 10.1039/d3tb01839c (PMC10650276; doi:10.1039/d3tb01839c)
Supplement: TB-011-D3TB01839C-s001 [file TB-011-D3TB01839C-s001.pdf]

Electronic Supplementary Material (ESI) for Journal of Materials Chemistry B.
This journal is © The Royal Society of Chemistry 2023

SUPPLEMENTAL INFORMATION

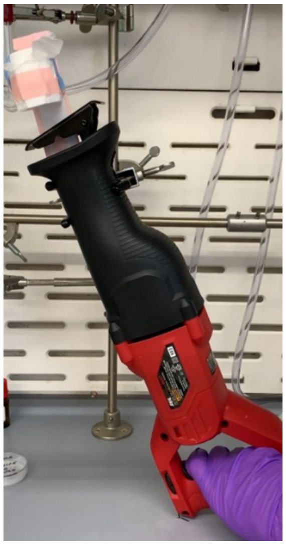

SI Figure 1. Image of custom emulsion mixer used to mix the aqueous phase into the organic phase of the emulsion.

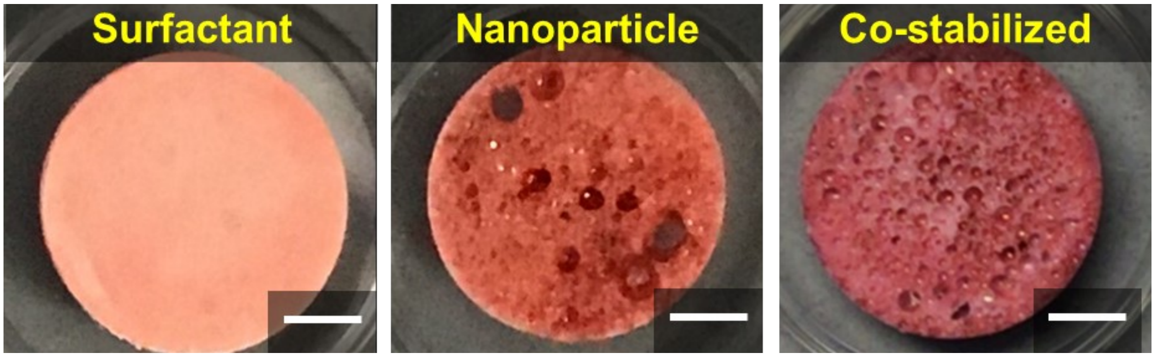

SI Figure 2: The presence of calcium is verified with Alizarin Red S. staining of nHA at the surface of the nHA-stabilized and co-stabilized porous scaffolds as compared to the negative control of the surfactant-stabilized porous scaffold. Scale bar = 2 mm.

SI Table 1: Gel fraction, compressive modulus, and compressive strength for surfactant-stabilized, nanoparticle-stabilized, and co-stabilized polyMIPEs.

	PGPR (n)	nHA (n)	Co-stabilized (n)
Gel Fraction (%)	96.6 ± 1.1 (8)	94.3 ± 1.7 (7)	97.5 ± 1.6 (9)
Compressive Modulus (MPa)	29.2 ± 6.4 (6)	44.9 ± 11.0 (5)	41.6 ± 6.4 (5)
Compressive Strength (MPa)	2.5 ± 0.4 (6)	3.9 ± 1.0 (5)	3.6 ± 0.5 (5)

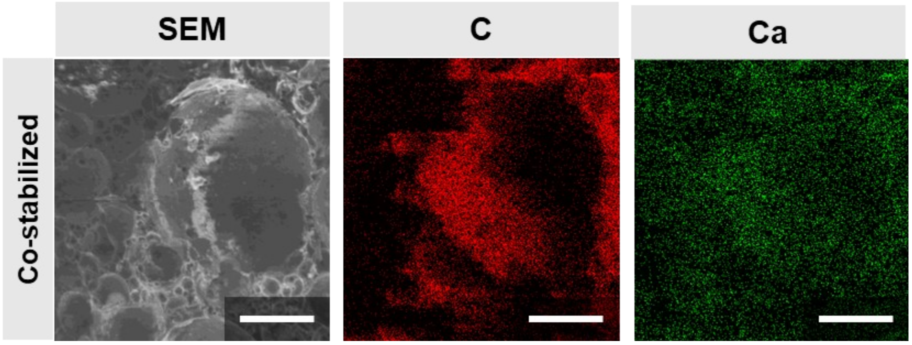

SI Figure 3: Carbon and calcium mapping with EDS to demonstrate the presence of nanoparticles at the pore surface of co-stabilized polyMIPEs. Scale bars = 100 µm.

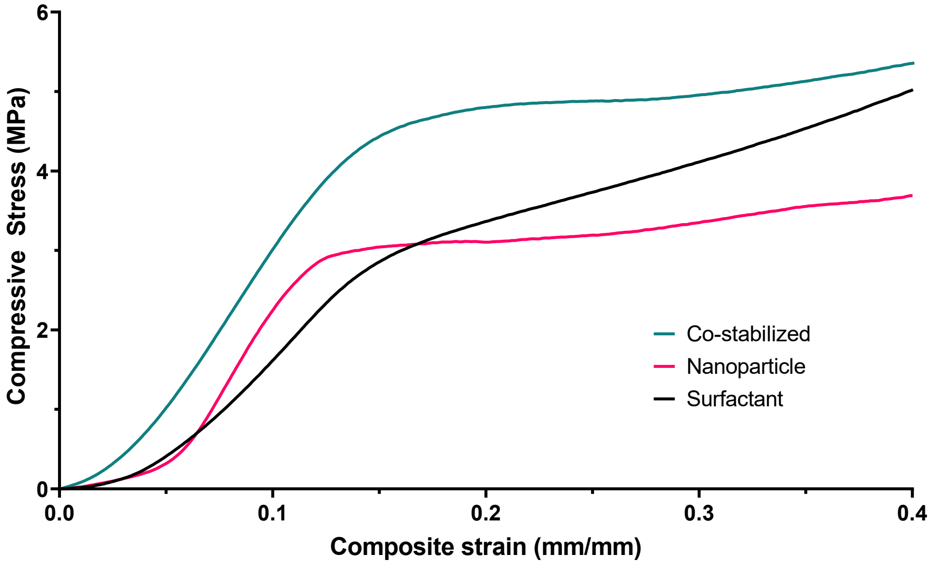

SI Figure 4: Representative stress-strain curves of surfactant, nanoparticle, and co-stabilized polyMIPEs.
